# Linc-OIP5 in the breast cancer cells regulates angiogenesis of human umbilical vein endothelial cells through YAP1/Notch/NRP1 signaling circuit at a tumor microenvironment

**DOI:** 10.1186/s40659-020-0273-0

**Published:** 2020-02-11

**Authors:** Qing Zhu, Jingchao Li, Qi Wu, Yongxia Cheng, Huizhe Zheng, Tao Zhan, Hongwei Wang, Yue Yang, Hongyan Wang, Ye Liu, Sufen Guo

**Affiliations:** 1 Department of Pathology, Mudanjiang Medical University, Mudanjiang, People’s Republic of China; 2 Department of Gynecology, Zhongda Hospital Lishui Branch Affiliated to Southeast University, Nanjing, People’s Republic of China; 3 Department of Neurology, Hongqi Hospital Affiliated to Mudanjiang Medical University, Mudanjiang, People’s Republic of China; 4 Department of Pathology, Hongqi Hospital Affiliated to Mudanjiang Medical University, Mudanjiang, People’s Republic of China; 5 Key Laboratory of Cancer Prevention and Treatment of Heilongjiang Province, Mudanjiang Medical University, Mudanjiang, People’s Republic of China

**Keywords:** Tumor microenvironment, Angiogenesis, Linc-OIP5, Signal transduction, Breast cancer, HUVECs

## Abstract

**Background:**

LincRNAs have been revealed to be tightly associated with various tumorigeneses and cancer development, but the roles of specific lincRNA on tumor-related angiogenesis was hardly studied. Here, we aimed to investigate whether linc-OIP5 in breast cancer cells affects the angiogenesis of HUVECs and whether the linc-OIP5 regulations are involved in angiogenesis-related Notch and Hippo signaling pathways.

**Methods:**

A trans-well system co-cultured HUVECs with linc-OIP5 knockdown breast cancer cell MDA-MB-231 was utilized to study the proliferation, migration and tube formation abilities of HUVECs and alterations of related signaling indicators in breast cancer cells and their conditioned medium through a series of cell and molecular experiments.

**Results:**

Overexpressed linc-OIP5, YAP1, and JAG1 were found in breast cancer cell lines MCF7 and MDA-MB-231 and the expression levels of YAP1 and JAG1 were proportional to the breast cancer tissue grades. MDA-MB-231 cells with linc-OIP5 knockdown led to weakened proliferation, migration, and tube formation capacity of co-cultured HUVECs. Besides, linc-OIP5 knockdown in co-cultured MDA-MB-231 cells showed downregulated YAP1 and JAG1 expression, combined with a reduced JAG1 level in conditioned medium. Furthermore, a disrupted DLL4/Notch/NRP1 signaling in co-cultured HUVECs were also discovered under this condition.

**Conclusion:**

Hence, linc-OIP5 in MDA-MB-231 breast cancer cells may act on the upstream of the YAP1/Notch/NRP1 signaling circuit to affect proliferation, migration, and tube formation of co-cultured HUVECs in a non-cellular direct contact way through JAG1 in conditioned medium. These findings at least partially provide a new angiogenic signaling circuit in breast cancers and suggest linc-OIP5 could be considered as a therapeutic target in angiogenesis of breast cancers.

## Background

Neovascularization is essential in some physiological and pathological processes, for instance, normal vasculature development, diabetic neovascularization, and tumor angiogenesis. Formation of new blood vessels can be regulated by some specific proteins, which all have functions in inducing proliferation, differentiation, and vascularization of endothelial cells (ECs) or endothelial progenitor cells (EPCs) [1–4]. The antiangiogenic therapy against tumor vasculature is currently one of the means to treat tumors. But recently, it has been found that the antiangiogenic therapy targeting vascular endothelial growth factor (VEGF) alone seems to be resistant. It is then particularly important to further study tumor-related angiogenic pathways.

Notch signaling pathway, as a highly conserved intercellular signaling pathway, is found to participate in several biological processes to control cell fate and tissue differentiation, including the normal vasculature development and angiogenesis [5–7]. Both Delta-like 4 (DLL4) and Jagged 1 (JAG1) are key ligands of the Notch pathway [1–3]. Recent evidence showed that they have opposite effects on regenerative vessel formation [1, 3]. DLL4 activates Notch signaling in the stalk ECs to inhibit sprouting angiogenesis and results in fewer but larger vessels, whereas JAG1 mainly increases sprouting angiogenesis and enhances the amount rather than the size of vessels by signaling to tumor cells and ECs [1–3, 8–16]. Both of them promote the growth of tumors, but different downstream signaling pathways activated by DLL4 and JAG1 lead to distinct vasculature phenotypes [1, 8, 17–19].

Neuropilin-1 (NRP1) is a transmembrane co-receptor that binds to VEGF family members and can enhance VEGF-A/VEGFR2 signaling via interactions between synectin [20–22]. It has been shown that in epidermal cancer stem (ECS) cells, NRP1 knockout could lead to the formation of small tumors characterized by decreased vascularization [23]. Besides, NRP1 has also been found to work in the downstream of the DLL4/Notch signaling pathway during angiogenesis [3] and there is a negative correlation between DLL4 and NRP1.

The Hippo signaling pathway is an evolutionarily conserved signaling cascade that regulates cellular proliferation and organ size [24–26]. It is controlled by the phosphorylation of downstream transcriptional coactivator Yes-associated protein (YAP) [26, 27]. Previous studies have shown that YAP could bind with the TEA domain (TEAD1) transcription factor to stimulate angiogenesis of ECs [28]. VEGF-A interacting with NRP1 could regulate Hippo signaling to drive tumor cell survival, angiogenesis, and tumor formation through facilitating the stabilization of YAP1 [23]. It is also shown that a direct transcriptional target of YAP1 is Notch signaling receptor Notch2, and Notch signaling is one of the downstream effectors of the Hippo signaling pathway [5, 26]. Reciprocally, YAP1 upregulates JAG1 expression and Notch signaling facilitates YAP1 activity and stability, indicating that there is a positive feedback loop between the YAP1 and JAG1/Notch signaling. This positive bidirectional circuit is functionally required for liver cell development and tumorigenesis [5, 26, 29].

Nowadays, several long noncoding RNAs (lncRNAs) have been strongly implicated in various cancers [30, 31]. As an isoform of lncRNAs, long intervening noncoding RNAs (lincRNAs) as transcript units between protein-coding genes have showed distinct tissue- or cell-specific expressions [32, 33]. Accumulating evidences indicated that abnormal expression of lincRNAs has been strongly correlated to tumor initiation, progression, and metastasis [34, 35]. Among them, a novel linc-Opa interacting protein 5 (linc-OIP5) has been revealed to be dysregulated in cancers [32, 36]. Deng et al. demonstrated that linc-OIP5 knockdown inhibited the cell proliferation, migration, and invasion in lung adenocarcinoma [32]. Additionally, linc-OIP5 certainly contributed to carcinogenic potential in multiple myeloma by controlling cell proliferation and apoptosis [36]. It seemed that linc-OIP5 functioned to promote tumorigenesis and progression, but the significance of linc-OIP5 involved in tumor angiogenesis was largely unknown. Here, we hypothesized that linc-OIP5 may regulate the angiogenesis of endothelial cells at a tumor microenvironment and mainly investigated that whether linc-OIP5 in breast cancer cells affects the angiogenesis of human umbilical vein endothelial cells (HUVECs) and whether this regulation is involved in angiogenesis-related YAP1/Notch/NRP1 signaling circuit.

## Materials and methods

### Clinical specimens

Sixty-seven cases of breast cancers were collected from the Department of Pathology of the Affiliated Hospital of Mudanjiang Medical University from 2015 to 2017. All patients were diagnosed based on the World Health Organization diagnostic criteria of mammary tumors. All patients had written informed consent and consent to use excess pathological specimens for research purposes. The clinicopathological variables of patients are summarized in Table 1. In addition, this research was approved by the Ethical Committee of Affiliated Hospital of Mudanjiang Medical University. Breast cancer tissues were immediately fixed with formalin for further use.

**Table 1:** Clinicopathological variables of patients with breast cancer (N = 67)

Clinicopathological variables	N
Age	
≤ 50	29
> 50	38
Menstrual status	
Before menopause	34
After menopause	33
Tumor size	
≤ 2.0 cm	36
> 2.0 cm	31
Lymphatic metastasis	
Be	28
No	39
Pathological grading	
Grade I	30
Grade III	37
TNM stages	
Stage I	35
Stage II	19
Stage III–IV	13

### Cell lines and culture conditions

Human breast cell lines (MCF-10 cells), Human breast cancer cell lines (MDA-MB-231 cells, MCF-7 cells) and Human umbilical vein endothelial cells (HUVECs) were purchased from Shanghai Cell Bank of Chinese Academy of Sciences. These cells were cultured in Dulbecco’s Modified Eagle Medium (DMEM) (Invitrogen; Gibco; 12800017) supplemented with 10% FBS (Gibco; 16000-044) and 1% penicillin–streptomycin (Hyclone; SV30010). Cells were incubated in a humidified atmosphere at 37 °C with 5% CO_2_.

### Immunohistochemistry

Paraffin-embedded breast cancer tissues were cut into 3 μm sections, followed by heating for 1 h at 60 °C. The slides were rehydrated for antigen retrieval, then treated with 0.01 M sodium citrate buffer (pH 6.0) boiled for 2 min at 100 °C. The samples were pretreated with 5% BSA for 30 min to block antibody nonspecific staining and then incubated with the indicated antibodies for 1 h at 37 °C. The sections were treated with 3% H_2_O_2_ for 10 min to block endogenous peroxidase activity, then incubated with biotinylated goat anti-rabbit IgG and horseradish peroxidase labeled avidin for 15 min at room temperature. Followed by DAB color development, the sections were counterstained with hematoxylin and sealed with neutral gum. Following primary antibodies were used for immunohistochemistry: antibodies against YAP1 (1:200; Gene Tex; GTX129151) and JAG1 (1:100; Gene Tex; GTX48691). Secondary antibodies used were: Rabbit SP Kit (ZSGB-BIO; SP-9001), followed by using DAB Substrate Kit (ZSGB-BIO; ZLI-9018). The sample images were captured by the Leica DM 1000 microscope from three different fields of view at magnification 200×. The presented images were representative and each one had at least three independent repetition.

### Reverse transcription PCR (RT-PCR) and quantitative real-time PCR (qRT-PCR)

Total RNA from cultured cells was isolated by Trizol reagent (OMEGA miRNA Kit; R6842-01) according to the manufacturer protocol, and cDNA was synthesized by reverse transcription using a Reverse Transcription Kit (Roche; 04897030001) and then amplified with FastStart Universal SYBR Green Master (ROX) kit (Roche; 04913850001). The relative abundance of mRNA was calculated using GAPDH mRNA as normalization. All primers for RT-PCR and qRT-PCR were purchased from Sangon Biotech (Shanghai Sangon). The following sequences of primer pairs were used to detect the mRNA levels of the indicated genes:

- Human Linc-OIP5 (forward primer 5′-GCTGCGAAGATGGCGGAGTAAG-3′ and reverse primer 5′-CACGGTCCAACAGATGCACTCG-3′);
- Human YAP1 (forward primer 5′-CCTGCGTAGCCAGTTACCAACAC-3′ and reverse primer 5′-GCTGCTCATGCTTAGTCCACTGTC-3′);
- Human JAG1 (forward primer 5′-TGTGGCTTGGATCTGTTGCTTGG-3′ and reverse primer 5′-ACGTTGTTGGTGGTGTTGTCCTC-3′);
- Human GAPDH (forward primer 5′-CAGGAGGCATTGCTGATGAT-3′ and reverse primer 5′-GAAGGCTGGGGCTCATTT-3′).

The images afterwards were sequentially scanned with a gel imaging system (Bio-Rad; 170-8195).

### Immunoblot

Cells were lysed in RIPA Lysis Buffer supplemented with protease inhibitors (Beijing Applygen; C1053). The protein concentrations were quantified with BCA Protein Assay Kit (Beijing Applygen; P1511). Equivalent protein quantities were subjected to SDS-PAGE gels and then transferred to PVDF membranes (Millipore; ISEQ00010). Membranes were then blocked with 5% non-fat milk in TBST for 1 h at room temperature and incubated overnight with indicated primary antibodies. On the following day, the PVDF membranes were washed at least three times for 5 min by TBST and incubated with the appropriate HRP-conjugated Affinipure Goat Anti-mouse/rabbit IgG (Proteintech; SA00001-1/SA00001-2) secondary antibodies for 1 h at room temperature. Immunoreactive bands were visualized with an enhanced chemiluminescence kit (Biosharp; BL520A). GAPDH and β-actin were used as protein loading controls. The following antibodies were used: antibodies against Notch1 (1:2000; Abcam; ab52627), NRP1 (1:1000; Abcam; ab81321), DLL4 (1:2000; Gene Tex; GTX109649), YAP1 (1:200; Gene Tex; GTX129151), JAG1 (1:100; Gene Tex; GTX48691), GAPDH (1:1000; Cloud-Clone Corp; RPB932Hu01), β-actin (1:800; Cloud-Clone Corp; RPB340Mi01). The Image-pro plus software was used for following densitometric analyses of Immunoblot. The quantification results were normalized by loading control.

### Small interfering RNAs

The small interfering RNAs (siRNAs) duplexes targeting linc-OIP5 and the negative control siRNA duplexes were synthesized and purchased from GenePharma (Shanghai GenePharma). The siRNA sequences were as follow:

- Negative control (NC) siRNA duplexes sense (5′-UUCUCCGAACGUGUCACGUTT-3′ and antisense 5′-ACGUGACACGUUCGGAGAATT-3′);
- siLinc-OIP5 Duplex1 sense (5′-CCUACUGCCUUGUAAGAAUTT-3′ and antisense 5′-AUUCUUACAAGGCAGUAGGTT-3′);
- siLinc-OIP5 Duplex2 sense (5′-CCAGCUGUCUUUGUGUCUUTT-3′ and antisense 5′-AAGACACAAAGACAGCUGGTT-3′);
- siLinc-OIP5 Duplex3 sense (5′-CCAGUUAUCCUGCUAACAUTT-3′ and antisense 5′-AUGUUAGCAGGAUAACUGGTT-3′).

This study used the mixtures of three siRNAs. Plasmids were transfected into tumor cells through Lipofectamine^®^3000 Transfection Reagent (Invitrogen Life Technologies; L3000008) according to the manufacturer instructions. Cells were seeded at a concentration of 2.8 × 10^5^ cells per well in 6-well plates. The effectiveness of knockdown via the above siRNAs was assessed by qRT-PCR.

### Enzyme-linked immunosorbent assay (ELISA)

JAG1 levels were detected in cultured supernatants of MDA-MB-231 cells and MCF-7 cells with a JAG1 specific ELISA kit (Cloud-Clone Corp; SEB807Hu 96T). Incubations were terminated before centrifugation at 1000×*g* for 20 min at 4 °C to remove cellular debris and then the supernatants were collected. ELISA assay determined the concentration of secreted JAG1 in tumor cells with or without linc-OIP5 siRNA according to the manufacturer instructions. The absorbance was measured at 450 nm using a SoftMax Pro microplate reader and the optical density values of each well represented the JAG1 levels in distinct samples. All of these experiments were performed in an independent way and repeated at least three times.

### Cell proliferation assay

HUVECs were collected at 48 h after cocultivation with MDA-MB-231 cells. Cell Counting Kit-8 (CCK-8) (Dojindo; CK04-500T) was used according to the manufacturer instructions. Cells were seeded in 96-well plates at the density of 4 × 10^3^ cells per well and CCK-8 reagents (10 μl/well) were added into the medium without serum (90 μl/well), followed by incubating for 3 h at 37 °C. The amount of formazan dye generated by cellular dehydrogenase redox was measured through absorbance at 450 nm, using a SoftMax Pro microplate reader. And the produced amount was proportional to the number of living cells. The cell proliferation was measured every 24 h for 4 days and the optical density values of each well represented the survival/proliferation cells ratio. These experiments were also performed independently and repeated at least three times.

### Cell migration assay

The wound-healing assay was used to analyze the migration ability of HUVECs after cocultivation with MDA-MB-231 cells. Cells (3 × 10^5^ HUVECs per well) were seeded on the lower chamber of a 24-well trans-well cell culture chamber and incubated at 37 °C in 5% CO_2_. Cells were then monitored for 48 h to permit cell adhesion and formation of confluent monolayers, which would be scratched using the tip of a p10 pipet afterwards. The scratched wound should be rinsed twice with PBS to remove the debris and then MDA-MB-231 cells were added on the upper chamber at a density of 6 × 10^4^ per well. The cells were incubated at 37 °C in 5% CO_2_ and monitored for 24 h. The wound could be healed during monitoring digital images at 0 h, 12 h, and 24 h after scratching and the images were captured from three different fields of three independent samples at magnification 40× using an inverted microscope (Nikon; TE2000-S). The extent of wound healing was assessed by the ratio of healing area to initial wound (0 h):

$${\text{R}}_{\text{n}} = \frac{{{\text{A}}_{ 0} - {\text{A}}_{\text{n}} }}{{{\text{A}}_{ 0} }} \times 100\%$$

R_n_ represents the percentage of wound closure, A_n_ represents the residual area of the wound at metering point (n h), and A_0_ represents the area of initial wound (0 h).

### Tube formation assay

HUVECs (2 × 10^5^) were seeded on each lower chamber of a 24-well trans-well cell culture chamber precoated with 150 μl Matrigel (BD Biosciences; 356234). The HUVECs were allowed to adhere for 30 min before seeding MDA-MB-231 cells on the upper chamber at a density of 4 × 10^4^ per well. For experimental treatments, HUVECs were incubated in 600 μl medium with 10% fetal bovine serum and co-cultured with MDA-MB-231 cells previously treated with or without linc-OIP5 siRNA. HUVECs were cultured for 6 h with different treatments to evaluate the formation of capillary-like tube structures. Images were captured from three different fields of three independent samples at magnification 4× using an inverted microscope (Nikon; TE2000-S). Tube analysis was performed via ImageJ software.

### Statistics and repeatability of experiments

Data were presented as mean ± standard deviation (s.d) and all error bars indicate s.d. SPSS software 21.0 and Graphpad Prism 7.0 were used to evaluate the statistical significance. The analysis of variance (one-way ANOVA) test was used to compare mean values among three or more data sets and the Student–Newman–Keuls was performed to compare the mean of each data with the mean of every other data. Non-parametric Tamhane T2 comparison was also employed. Furthermore, Statistical comparisons of means were made using the unpaired Student’s two-tailed t-test for two data sets. For statistical tests, *P* < 0.05 was used as the criterion for statistical significance. The independent experiments were repeated at least three times.

## Results

### YAP1 and JAG1 were overexpressed in breast cancer cells and tissues

**Fig. 1:**
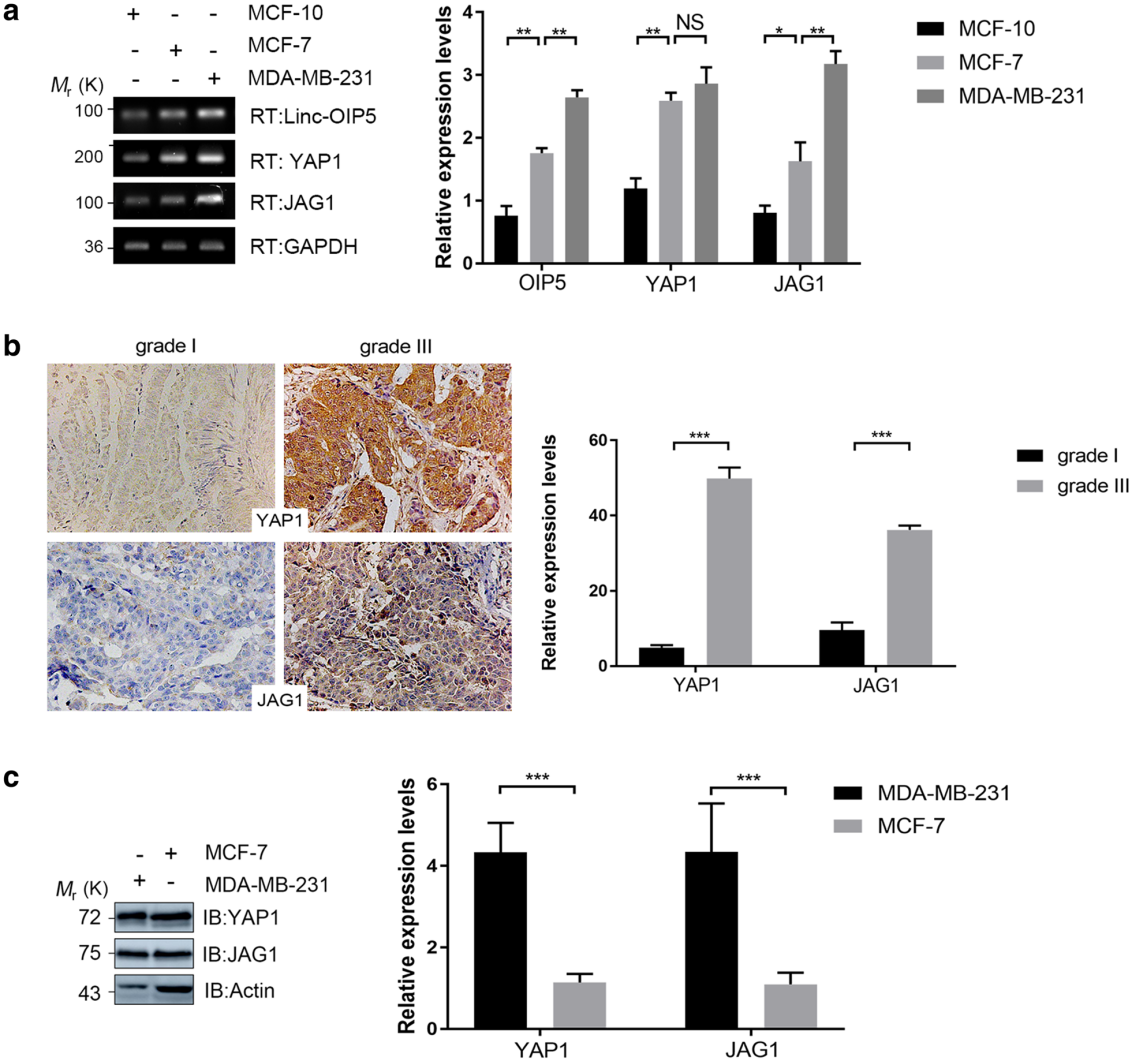
Expressions of YAP1 and JAG1 in breast cancer cells and tissues were upregulated. **a** RT-PCR and its quantification were used to determine the mRNA levels of linc-OIP5, YAP1 and JAG1, which were differentially expressed in normal and cancerous breast cells. **b** Immunohistochemistry analysis of breast cancer tissue samples confirmed that YAP1 and JAG1 are significantly higher in grade III than grade I and YAP1 in cancer tissues also displayed robust nuclear localization. **c** Immunoblot analysis and its quantification were performed to check the expression levels of YAP1 and JAG1 in MDA-MB-231 and MCF-7 cells. Fold changes were obtained by normalizing against β-actin. Graphs are means of indicated stained area in 5 samples and error bars indicate s.d. Magnification×200. *P < 0.05, ** *P *< 0.01, ***P < 0.001

In order to investigate the role of linc-OIP5, YAP1, and JAG1 in breast tumorigenesis, their expression levels in normal breast cells (MCF-10) and breast cancer cells (MDA-MB-231, MCF-7) were verified by RT-PCR. Compared with normal breast MCF-10 cells, expression levels of them were obviously higher in MDA-MB-231 and MCF-7 cells (Fig. 1a), indicating that these molecules were all positively correlated with the development of breast cancer. What’s more, their expression levels in MDA-MB-231 cells were higher than that in MCF-7 cells (Fig. 1a).

Furthermore, immunohistochemistry analysis of breast cancer tissues showed that YAP1 and JAG1 were differentially expressed in breast cancers with different grades. The expression degrees of these two proteins were proportional to the grade of cancer tissues according to the World Health Organization diagnostic criteria (Fig. 1b). Besides the changes of YAP1 expression in breast cancer tissues, it also displayed robust nuclear localization in which it is activated (Fig. 1b). The immunoblot analysis further confirmed the protein expression of YAP1 and JAG1 in both breast cancer cells, which gave the same results as RT-PCR (Fig. 1c). Therefore, the results suggest that the expressions of YAP1 and JAG1 are upregulated in breast cancer cells and they may be associated with advanced tumor grade and poor prognosis for breast cancers.

### MDA-MB-231 cells were adopted to co-culture with HUVECs

**Fig. 2:**
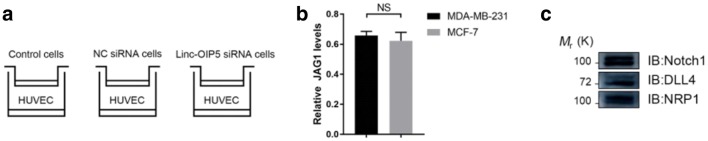
MDA-MB-231 cells were adopted to co-culture with HUVECs. **a** Cell co-culturing simulations: the MDA-MB-231 cells were co-cultured with HUVECs in indirect cell–cell contacts by a trans-well system. **b** Quantification of JAG1 expression in the conditioned medium of MDA-MB-231 and MCF7 cells. **c** Immunoblot results of the expression levels of DLL4, Notch1 and NRP1 in HUVECs. *NS* no statistical difference

To study the effects of secretory products from breast cancer cells on HUVECs, a trans-well system was adopted to achieve a tumor microenvironment, in which breast cancer cells transfected with the linc-OIP5 siRNA were co-cultured with the HUVECs in an indirect cell–cell contact (Fig. 2a). To select a better breast cancer cell line in this experiment, ELISA was performed to verify the relative levels of JAG1 in conditioned medium of both breast cancer cells (MDA-MB-231, MCF-7), which showed that the JAG1 expression levels in MDA-MB-231 cells were slightly higher than that in MCF-7 cells (Fig. 2b). Combined with the above studies, MDA-MB-231 cells with higher-grade malignancy were selected for further experimental analysis. Besides, expression levels of DLL4, Notch1, and NRP1 in HUVECs before co-culturing were detected by immunoblot. The results revealed that DLL4, Notch1, and NRP1 were all strongly expressed in HUVECs (Fig. 2c).

### Linc-OIP5 knockdown in breast cancer cells suppressed proliferation and migration of HUVECs

**Fig. 3:**
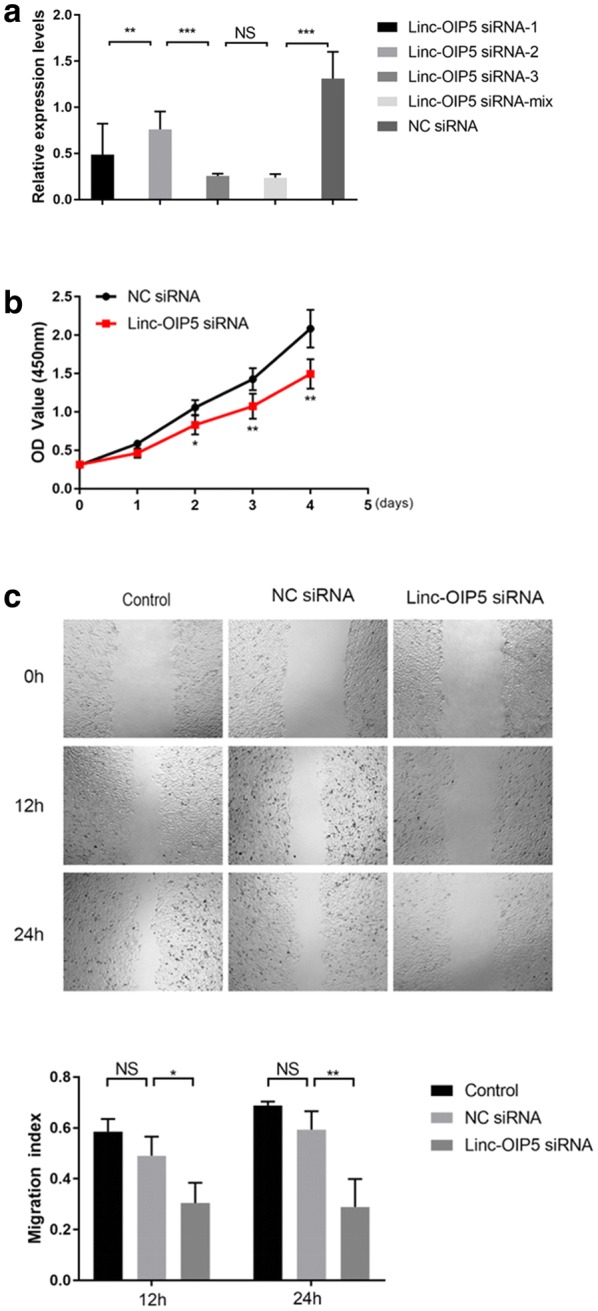
Knockdown of linc-OIP5 in MDA-MB-231 cells suppressed the proliferation and migration of co-cultured HUVECs in vitro. **a** Relative expression levels of linc-OIP5 were detected after MDA-MB-231 cells transfected with linc-OIP5 siRNAs. **b** Knockdown of linc-OIP5 significantly suppressed HUVECs proliferative capacity by CCK-8 assays. **c** Migration ability of the co-cultured HUVECs after linc-OIP5 knockdown reduced appreciably through wound healing assays. Fold changes were obtained by normalizing against control group. Magnification×40. *NS* no statistical difference, *P < 0.05, ** *P *< 0.01

As linc-OIP5 was also upregulated in the breast cancer cells as aforementioned (Fig. 1a), three linc-OIP5 siRNAs were adopted to achieve linc-OIP5 knockdown in the MDA-MB-231 cells. The transfection efficiency of all three siRNAs and their mixture was assessed, which showed that the mixture of linc-OIP5 siRNAs contained the highest knockdown effect in the MDA-MB-231 cells (Fig. 3a). Furthermore, MDA-MB-231 cells transfected with linc-OIP5 siRNA (mixture) showed inhibited cell proliferation and migratory ability of its co-cultured HUVECs (Fig. 3b, c). These findings suggest that MDA-MB-231 cells with linc-OIP5 knockdown suppress the proliferation and migration of their co-cultured HUVECs.

### Linc-OIP5 in breast cancer cells suppressed tube-formation ability of co-cultured HUVECs

**Fig. 4:**
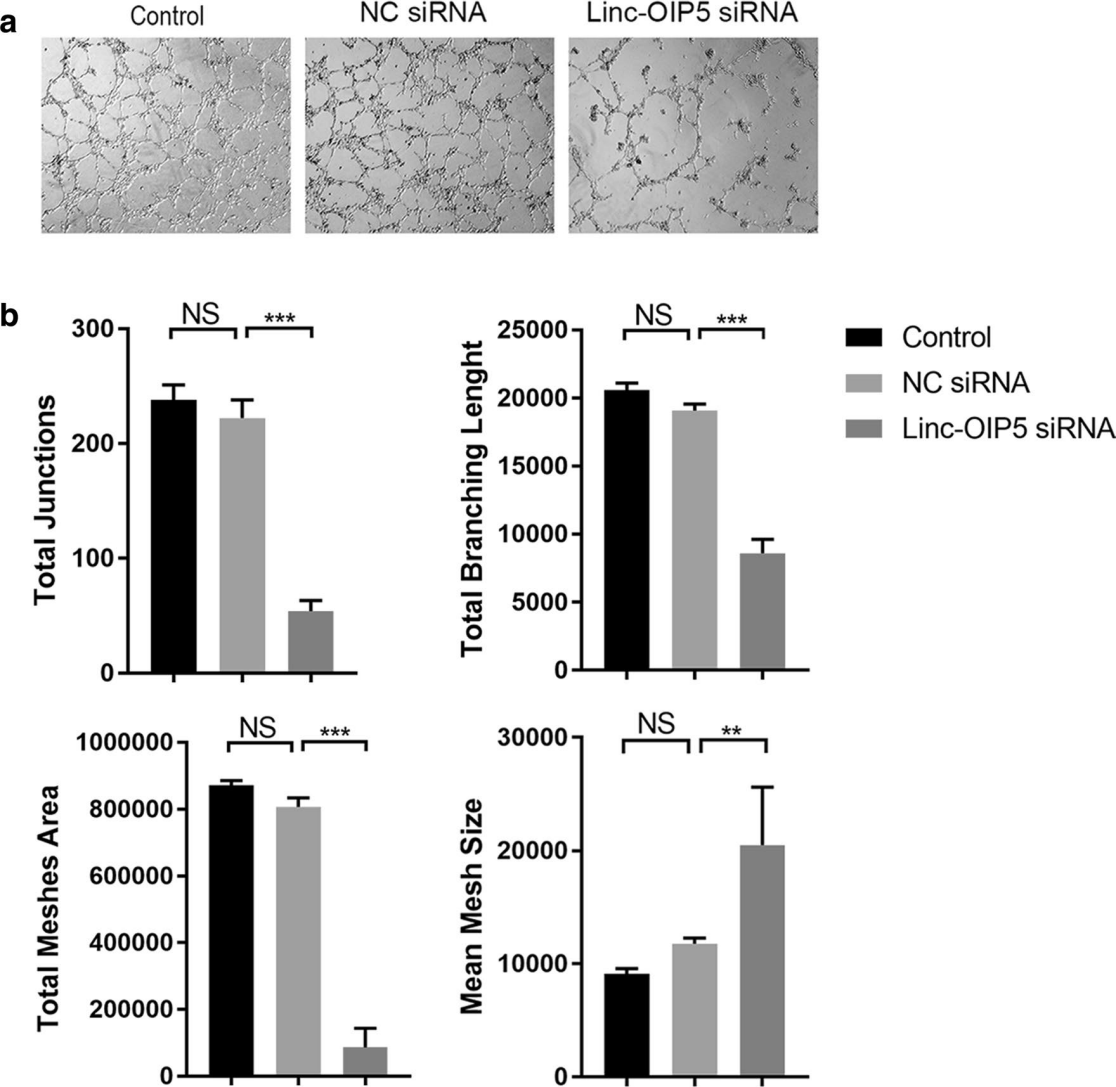
Linc-OIP5 knockdown in MDA-MB-231 cells affected angiogenesis capacity of HUVECs in vitro. **a** HUVECs capillary-like tube formation assay indicated that linc-OIP5 downregulation dramatically reduced the angiogenesis of co-cultured HUVECs. Magnification×40. **b** Tube formation assay analysis was quantified by the ImageJ software, plotted as the number of total junctions, the lenght of total branching, the area of total meshes, and the size of mean mesh. Fold changes were obtained by normalizing against control group. *NS* no statistical difference, ** *P *< 0.01, ***P < 0.001

Subsequently, HUVECs capillary-like tube formation as a surrogate model of angiogenesis was investigated. After co-culturing for 6 h, tube formation was observed microscopically and images were statistically evaluated. It was showed that the amount of vessel formation in co-cultured HUVECs was largely decreased (Fig. 4a). Statistically, the junctions between vessels were reduced dramatically after linc-OIP5 knockdown, which was consistent with the less branching length in this group. Meanwhile, the mean mesh size between vessels was the largest in linc-OIP5 knockdown cells, but the total mesh area was opposite which could result from the fewer vessels induced by linc-OIP5 knockdown in breast cancer cells (Fig. 4b). Overall, these figures indicate that linc-OIP5 knockdown in MDA-MB-231 cells suppresses the HUVECs capillary-like tube formation at a tumor microenvironment.

### Linc-OIP5 knockdown decreased YAP1 and JAG1 expression levels at a breast cancer microenvironment

**Fig. 5:**
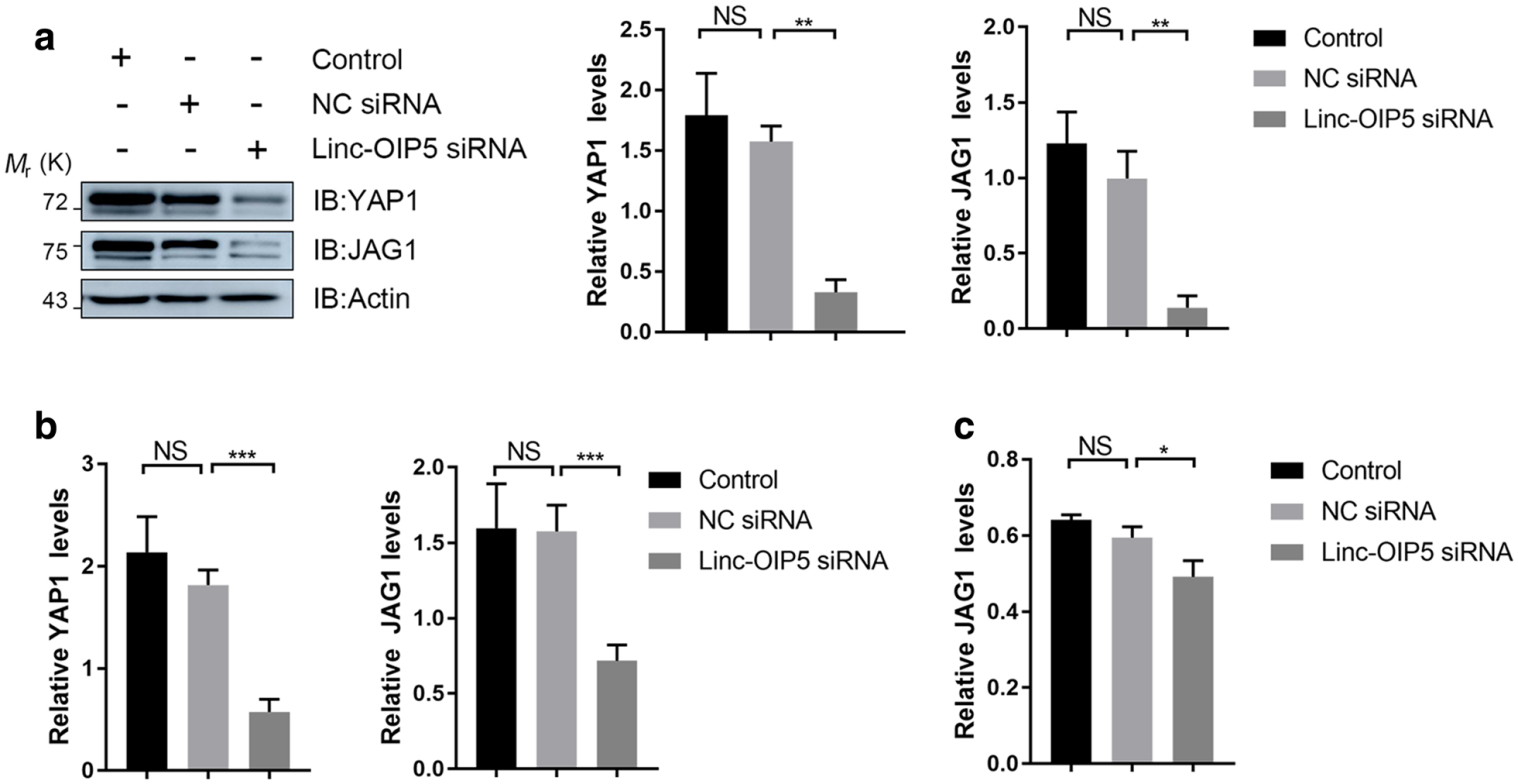
Downregulation of YAP1 and JAG1 was observed in MDA-MB-231 cells with linc-OIP5 knockdown. **a** Immunoblot confirmed linc-OIP5 siRNA remarkably decreased the protein expressions of YAP1 and JAG1. **b** qRT-PCR was used to check the mRNA levels of YAP1 and JAG1 after linc-OIP5 knockdown. **c** Linc-OIP5 siRNA decreased the JAG1 levels in the conditioned medium of MDA-MB-231 cells by ELISA analysis. *NS* no statistical difference, *P < 0.05, ** *P *< 0.01, *** *P *< 0.001

To explore whether the regulation of HUVEC tube formation ability by linc-OIP5 was associated with angiogenesis-related YAP1/Notch/NRP1 signaling pathway, the expression levels of YAP1 and JAG1 in MDA-MB-231 cells were investigated. Immunoblot results showed that linc-OIP5 knockdown remarkably downregulated the protein levels of YAP1 and JAG1 in the cells (Fig. 5a). Consistently, the mRNA expression levels of YAP1 and JAG1 in the linc-OIP5 knockdown cells were also dramatically decreased with significant differences (Fig. 5b). Besides, further ELISA showed that linc-OIP5 knockdown decreased the JAG1 protein levels in the conditioned medium of MDA-MB-231 cells (Fig. 5c). These results inform that linc-OIP5 knockdown decreases the expression of both YAP1 and JAG1 in MDA-MB-231 cells and JAG1 may act as an intermediate to link linc-OIP5 related signaling with vessel formation of HUVECs through an indirect way at a breast cancer microenvironment.

### Linc-OIP5 knockdown in MDA-MB-231 cells disrupted proangiogenic signaling in HUVECs

**Fig. 6:**
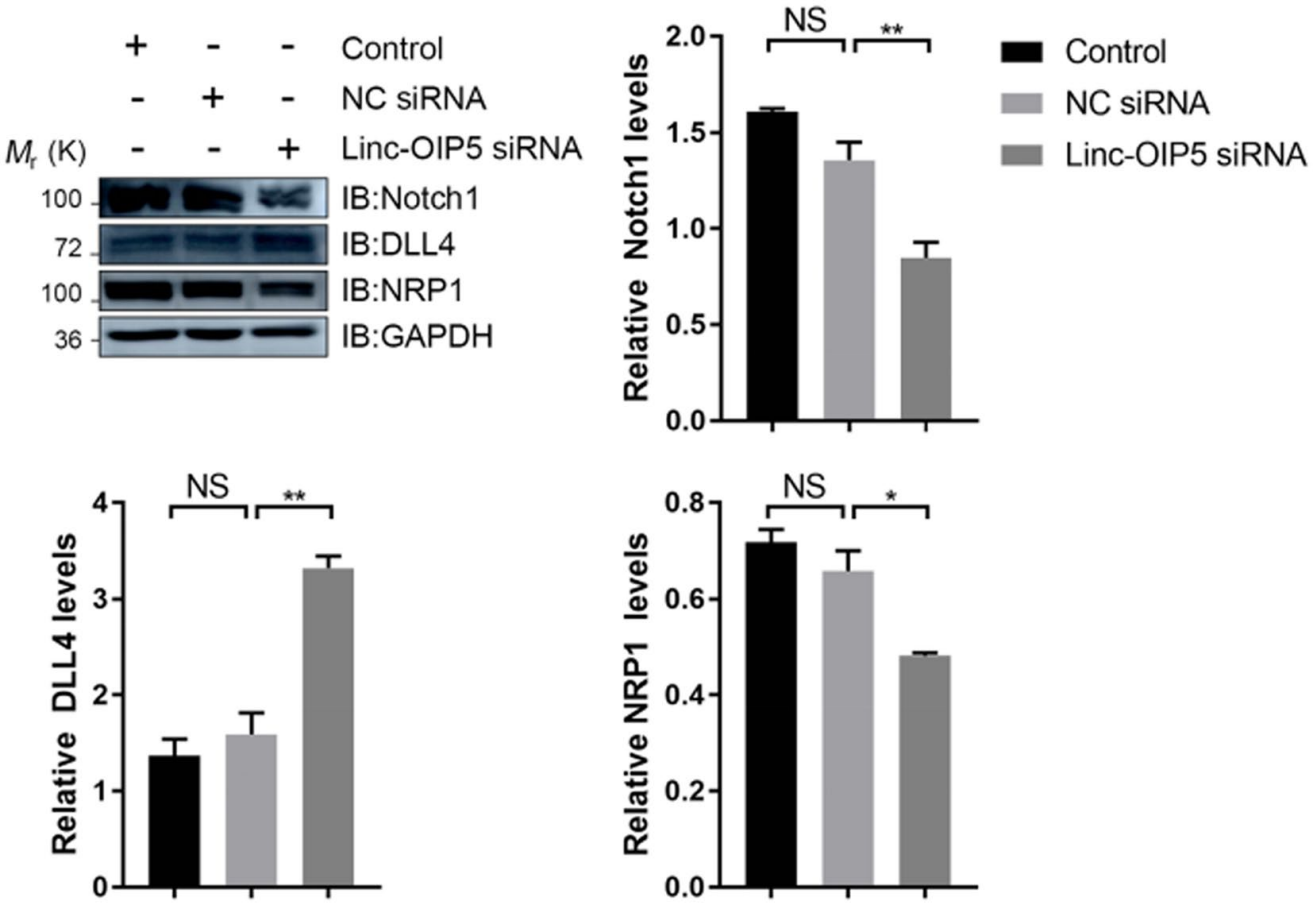
Linc-OIP5 knockdown in MDA-MB-231 cells disrupted the Notch1, DLL4 and NRP1 expressions in co-cultured HUVECs. The Notch1, DLL4 and NRP1 expressions were detected by immunoblot using the indicated antibodies. Fold changes were obtained by normalizing against control group. *NS* no statistical difference, *P < 0.05, ** *P *< 0.01

Furthermore, we also determined the expression levels of some proangiogenic agents, like DLL4, Notch1, and NRP1 in the HUVECs when they were co-cultured with linc-OIP5 knockdown MDA-MB-231 cells through immunoblot. As shown in Fig. 6, compared with the control, the expression levels of DLL4 was increased largely, while the relative expressions of Notch1 and NRP1 had a significant decrease (Fig. 6). These findings suggest that linc-OIP5 knockdown disrupts the normal proangiogenic signaling in co-cultured HUVECs with abrogating direct apposition at a breast cancer microenvironment.

## Discussion

Existing studies showed that linc-RNAs are considered as tumor enhancers and are closely correlated to tumor initiation, progression, and metastasis [34, 35]. Linc-OIP5 has been revealed to have carcinogenic potentials in lung adenocarcinoma and multiple myeloma [32, 36]. Here, we demonstrated a functional role of the linc-OIP5 in MDA-MB-231 cells to regulate tube formation abilities of HUVECs at a co-cultured microenvironment through the YAP1/Notch/NRP1 signaling circuit. Linc-OIP5 knockdown in the MDA-MB-231 cells suppressed the proliferation activity, migration and tube formation capacity of co-cultured HUVECs. Furthermore, the expression of YAP1 and its downstream regulator JAG1 in MDA-MB-231 cells were decreased a lot after linc-OIP5 knockdown. Through reduced level of JAG1 in the conditioned medium of MDA-MB-231 cells with linc-OIP5 knockdown, it seemed that JAG1 may act as an intermediate to link the linc-OIP5-correlated signaling with further cell behaviors of HUVECs. Therefore, reduced JAG1 in the conditioned medium after linc-OIP5 knockdown may be one of the reasons to cause less blood vessel formation of HUVECs (Fig. 4). In addition, increased DLL4, with lower Notch1 and downstream NRP1 expressions in co-cultured HUVECs with linc-OIP5 knockdown MDA-MB-231 cells were observed. These factors are all the crucial regulators in the proangiogenic signaling. It was possible that the changes of these factors might be more or less affected by decreased JAG1 in conditioned medium and in turn, changed JAG1 in the conditioned medium maybe also influenced by DLL4/Notch/NRP1 signaling.

Previous studies demonstrated that the suppressed DLL4/Notch signaling promoted a highly branched network with small-caliber vessels, whereas increased activity encourages a sparse network of large-caliber vessels in ECs [1, 2]. On the contrary, the JAG1 has an opposite effect with DLL4 on regenerative vessels formation [1–3]. The DLL4 overexpression showed fewer but larger vessels whereas JAG1 upregulation produced more vessels in tumor cells [1]. Our results also showed a higher level of DLL4 in co-cultured HUVECs with a less amount of blood vessel formation when linc-OIP5 was knocked down in breast cancer cells, which was roughly consistent with previous studies, although the changes of the size of blood vessels were not obvious from our experiments. This need more experiments to verify. The sum of our results delineated that linc-OIP5 in breast cancer cells may act on the upstream of YAP1/Notch/NRP1 signaling circuit associated with tube formation abilities of HUVECs through an indirect cell–cell contact.

While the mechanism underlying linc-OIP5 regulated angiogenesis with abrogating direct apposition is not yet fully understood, it might be a probable way to facilitate the interaction between the signaling networks in cancer cells and angiogenesis in ECs at a tumor microenvironment. Along this line, paracrine actions play an important role in molecular crosstalk between the angiogenesis signaling circuit in tumor microenvironment, and exosomes are functional components of paracrine secretion [6, 7]. From our experiments, linc-OIP5 knockdown may decrease the JAG1 content in the exosomes of conditioned medium through paracrine actions, which indirectly affected correlative protein expressions and cell activity of co-cultured HUVECs.

Linc-OIP5 may have a certain relationship with tumor resistance and metastasis. In recent years, it was found that traditional antiangiogenic drugs can gradually increase tumor resistance and accelerate tumor metastasis, which is tightly associated with vasculogenic mimicry (VM) and marked hypoxia. We previously showed that the inhibition of Notch signaling promoted the trans-differentiation of glioma stem cells toward the endothelial cell and tumor vascularization. Additionally, HIF-1α could reduce Notch signaling to stimulate vascularization [37]. Our other study demonstrated that miR-34a induced the trans-differentiation of glioma stem cells and vascular formation by inhibiting Notch signaling [38]. Furthermore, Hippo signaling coactivator YAP regulates stem cells pluripotency and its phosphorylation is crucial to the biological function of HIF-1α [16, 39–41]. Therefore, Notch signaling and YAP are considered to be correlated with the VM and hypoxia, which can further affect tumor resistance and metastasis. As the role of linc-OIP5 in the upstream of YAP1/Notch signaling was confirmed by this study, antiangiogenic therapies targeting linc-OIP5 will be meaningful for tumor resistance and metastasis.

Moreover, vascular normalization (VN) is a new perspective of the antiangiogenic therapy targeting tumor vasculature recently. Evidence indicated that blockade of angiogenesis can delay tumor growth, but may also paradoxically accelerate tumor metastasis [42–44]. VN could solve this paradox [42]. From the perspective of VN, normal mature blood vessels increase blood perfusion in the tumor microenvironment. Besides promoting tumor growth, this phenomenon would also transfer the oxygen and drugs to tumor cells more effectively, thus enhancing the sensitivity of tumor tissues to radiotherapy and chemotherapy. Based on the theory and results in this study, linc-OIP5 played a crucial role in tumor angiogenesis. After knocking down linc-OIP5 in breast cancer cells, JAG1 expression was downregulated while DLL4 expression was upregulated in co-cultured HUVECs, rendering the production of fewer blood vessels. According to the existing experimental results, DLL4 could promote fewer and larger vessels, but the newly-formed vessels had poor maturity [1], which was still with gap in the expectation of VN. If linc-OIP5 can regulate DLL4 by cooperating with certain pro-vascular maturation factors to promote VN, it will have some considerable implications for tumor targeting therapy.

## Conclusions

This study demonstrated that linc-OIP5 knockdown in MDA-MB-231 cells suppressed the proliferation, migration and tube formation of co-cultured HUVECs by disrupting YAP1/Notch/NRP1 signaling circuit. With the help of effects from the possible intermediate JAG1 in the conditioned medium, linc-OIP5 could regulate YAP1/JAG1 signaling in breast cancer cells to further control DLL4/Notch/NRP1 signaling in co-cultured HUVECs, which maybe a novel mechanism of angiogenic regulations with abrogating direct apposition in a breast cancer microenvironment. Thus, linc-OIP5 can be considered as a suitable target for combinatory interventions that simultaneously perturbs these processes and may present promising strategies of potential synergy for breast cancer resistance and metastasis.

## Data Availability

The analyzed datasets during present study are available from the corresponding author on reasonable request.

## Abbreviations

ECs: Endothelial cells
EPCs: Endothelial progenitor cells
HUVECs: Human umbilical vein endothelial cells
VEGF: Vascular endothelial growth factor
DLL4: Delta-like 4
JAG1: Jagged 1
NRP1: Neuropilin-1
YAP: Yes-associated protein
lncRNAs: Long noncoding RNAs
lincRNAs: Long intervening noncoding RNAs
linc-OIP5: Linc-Opa interacting protein 5
VM: Vasculogenic mimicry
VN: Vascular normalization
